# Tortricid Moths Reared from the Invasive Weed Mexican Palo Verde, *Parkinsonia aculeata*, with Comments on their Host Specificity, Biology, Geographic Distribution, and Systematics

**DOI:** 10.1673/031.011.0107

**Published:** 2011-01-24

**Authors:** John W. Brown, Ricardo Segura, Quiyari Santiago-Jiménez, Jadranka Rota, Tim A. Heard

**Affiliations:** ^1^ Systematic Entomology Laboratory, Plant Sciences Institute, ARS, U.S. Department of Agriculture, National Museum of Natural History, Washington, DC 20013-7012; ^2^ CSIRO Ecosystem Sciences, Mexican Field Station, A. Carlón No. 5, Col. Ejido 1 de Mayo, Boca del Río C.P. 94297, Veracruz, Mexico; ^3^ Department of Entomology, National Museum of Natural History, Smithsonian Institution, Washington, DC 20013-7012; ^4^ CSIRO Ecosystem Sciences, Brisbane, Australia

**Keywords:** Australia, *Amorbia*, biological control, *Cochylis*, DNA sequences, host plants, Neotropics, *Ofatulena*, *Playtnota*, *Rudenia*, taxonomy

## Abstract

As part of efforts to identify native herbivores of Mexican palo verde, *Parkinsonia aculeata* L. (Leguminosae: Caesalpinioideae), as potential biological control agents against this invasive weed in Australia, ten species of Tortricidae (Lepidoptera) were reared from Guatemala, Mexico, Nicaragua, and Venezuela: *Amorbia concavana* (Zeller), *Platynota rostrana* (Walker), *Platynota helianthes* (Meyrick), *Platynota stultana* Walsingham (all Tortricinae: Sparganothini), *Rudenia leguminana* (Busck), *Cochylis* sp. (both Tortricinae: Cochylini), *Ofatulena duodecemstriata* (Walsingham), *O. luminosa* Heinrich, *Ofatulena* sp. (all Olethreutinae: Grapholitini), and *Crocidosema lantana* Busck (Olethreutinae: Eucosmini). Significant geographic range extensions are provided for *O. duodecemstriata* and *R. leguminana.* These are the first documented records of *P. aculeata* as a host plant for all but *O. luminosa.* The four species of Sparganothini are polyphagous; in contrast, the two Cochylini and three Grapholitini likely are specialists on Leguminosae. *Ofatulena luminosa* is possibly host specific on *P. aculeata.* Host trials with *Rudenia leguminana* also provide some evidence of specificity, in contrast to historical rearing records. To examine the possibility that *R. leguminana* is a complex of species, two data sets of molecular markers were examined: (1) a combined data set of two mitochondrial markers (a 781-basepair region of cytochrome c oxidase I (COI) and a 685-basepair region of cytochrome c oxidase II) and one nuclear marker (a 531-basepair region of the 28S domain 2); and (2) the 650-basepair “barcode” region of COI. Analyses of both data sets strongly suggest that individuals examined in this study belong to more than one species.

## Introduction

*Parkinsonia aculeata* L. (Leguminosae: Caesalpinioideae), commonly known as Jerusalem thorn, Mexican palo verde, and/or jellybean tree, is a large shrub or small tree native to North and South America that shows marked genetic divergence among populations (Hawkins et al. 2007). It is used as an ornamental in tropical and subtropical climates, and it has been used to re-vegetate desertified regions throughout the pantropics (van Klinken et al. 2008). It has escaped cultivation and become established in many places around the globe. It was introduced into Australia as an ornamental and shade tree around 1900 (Woods 1992) and by 1906 was considered weedy in some parts of Queensland (Bailey 1906). Now it is regarded as one of the most troublesome invasive weeds in northern Australia and is recognized as a Weed of National Significance. *Parkinsonia aculeata* currently infests over 800,000 hectares, mainly along watercourses, in Western Australia, Queensland, and the Northern Territory. It has the potential to invade most of the semi-arid to subhumid tropical areas in Australia (van Klinken et al. 2008).

*Parkinsonia aculeata* has been the target of biological control investigations by Australian researchers for nearly two decades (Woods 1992; Heard 2006). During initial exploration in Texas and northeastern Mexico that focused on the discovery of native herbivores of *P. aculeata*, members of six insect orders were recorded (Coleoptera, Diptera, Hemiptera, Lepidoptera, Orthoptera, and Thysanoptera) (Woods 1992). The five species of Lepidoptera reared during these efforts were *Brachyacma palpigera* (Walsingham) (Gelechiidae), *Anacamptodes* cf. *obliquaria* Grote (Geometridae), *Melipotis acontioides* (Guenée) (Noctuidae), *Carmenta* sp. (Sesiidae), and *Ofatulena luminosa* Heinrich (Tortricidae). More recently (1995–2009) additional species of Lepidoptera have been recorded from this weed pest. The purpose of this paper is to present records of the ten species of Tortricidae that have been reared from *Parkinsonia aculeata,* along with comments on their host range, damage, and geographic distribution. The results of host specificity trials for *Platynota stultana* Walsingham and *Rudenia leguminana* (Busck) are also presented. All of the material listed in the “Specimens Examined” sections was reared from *Parkinsonia aculeata* (unless stated otherwise) during the most recent ongoing study (1995–2009); other records of host-use and geographic distribution are from the literature and/or museum collections. Voucher specimens are deposited in the collections of the National Museum of Natural History (USNM), Smithsonian Institution, Washington, D.C., U.S.A.; and the Australian National Insect Collection (ANIC), Canberra, Australia. EME refers to Essig Museum of Entomology, University of California, Berkeley, U.S.A. LPL stands for Long Pocket Laboratories, CSIRO Entomology, Brisbane, Queensland, Australia; each reared specimen is assigned an LPL number for tracking.

## Species Accounts

**Figure 1–8.**
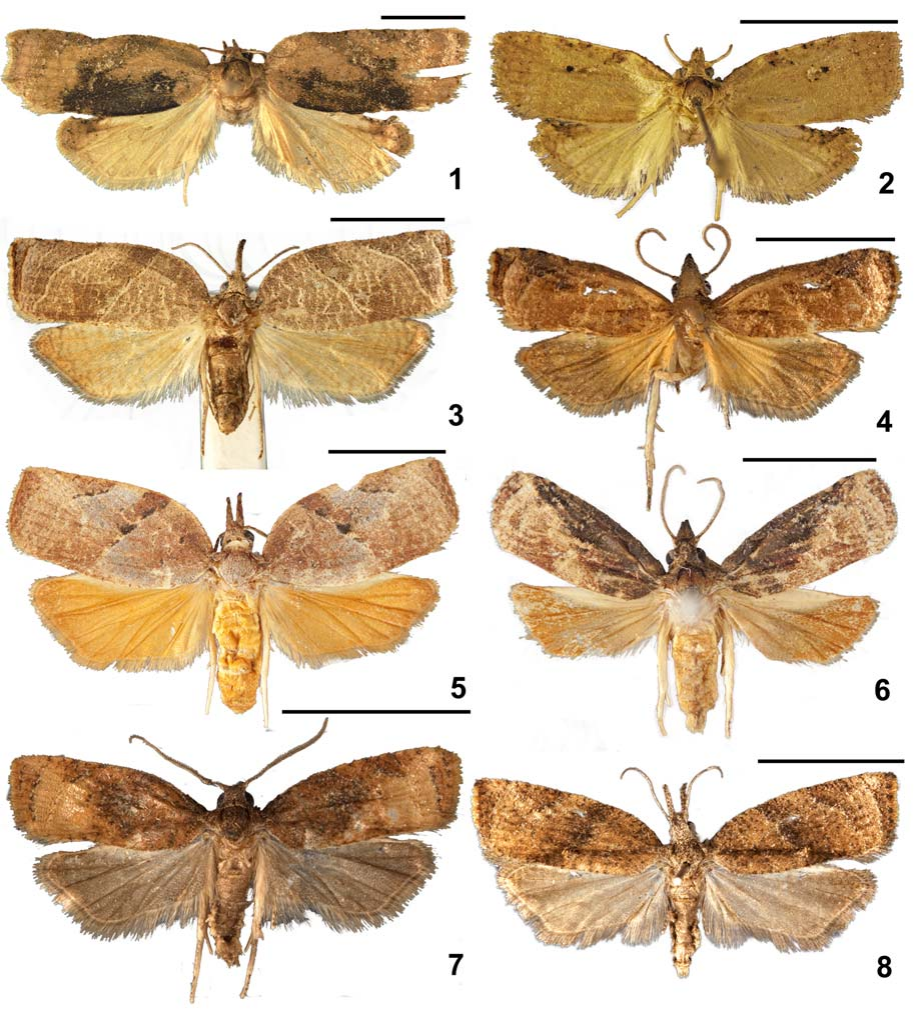
Adults of leaf-roller moths reared from *Parkinsonia aculeata.* 1) *Amorbia concavana* (Zeller) (♀). 2) *A. concavana* (♂). 3) *Platynota rostrana* (Walker) (♀). 4) *P. rostrana* (♂). 5) *Platynota subargentea* Walsingham (♀). 6) *P. subargentea* (♂). 7) *Platynota stultana* (♀). 8) *Platynota stultana* (♂). Scale bar = 5 mm. High quality figures are available online.

***Amorbia concavana* (Zeller) (Figures 1, 2)**
In a systematic revision of *Amorbia,* Phillips-Rodriguez and Powell (2007) recorded *A. concavana* from Costa Rica, Cuba, Guatemala, Honduras, Mexico, and Panama and provided illustrations of the adult and genitalia. Specimens that key to *Amorbia* *concavana* (Phillips-Rodriguez and Powell 2007) were reared from *P. aculeata* in Guatemala and Mexico. However, the specimens also resemble *A. emigratella* Busck, another widespread, polyphagous species of *Amorbia.*
**Biology.** Phillips-Rodriguez and Powell (2007) listed rearing records from *Mimosa pigra* L. (Leguminosae) in Mexico, *Phaseolus* sp. (Leguminosae) in Cuba, and *Inga vera* Willd. (Leguminosae) and *Hammelia* sp. (Rubiaceae) in Costa Rica. Although most recorded hosts are in Leguminosae, *A. concavana* likely is a polyphagous leaf-roller as are other members of the genus.
**Specimens Examined.** GUATEMALA: Jutiapa: Lago Guija, 14° 73.9′ N, 89° 32′ W, 14 January 2007 (1♀), R. Segura & M. Martínez, LPL 10955. MEXICO: San Luis Potosí: Laguna Ajinche, 22° 10.5′ N, 98° 21′ W, 18 May 2006 (1♂ 2♀), R. Segura & C. Pascacio, LPL 10688, 10887. La Marland, 22° 08.4′ N, 98° 24.9′ W, 9 November 2006 (1♂), R. Segura & M. Martínez, LPL 10897. Veracruz: Canoas, 22° 10.1′ N, 98° 07.7′ W, 16 August 2002 (6♂, 3♀), M. Martínez & C. Pascacio, LPL 12047, 12056.

***Platynota rostrana* (Walker) complex (Figures 3, 4)**
*Platynota rostrana,* which may represent a complex of closely related species, ranges throughout much of the southeastern U.S. and the northern Neotropics, including the Caribbean (based on specimens in USNM). Males are characterized by a complex hood of scaling on the frons of the head and a long costal fold that extends about 0.7 times the length of the forewing.
**Biology.** *Platynota rostrana* has been reared from more than 100 different plant species in over 20 different families (Heinrich 1921; MacKay 1962; d'Araujo Silva et al. 1968; Okumura 1974; Bruner et al. 1975; Diniz and Morais 1995; McClay et al. 1995). Examples of this species were reared from *P. aculeata* in Veracruz and San Luis Potosí, Mexico.
**Specimens Examined.** MEXICO: San Luis Potosí: Playa Buda, 22° 10.8′ N, 98° 22.8′ W, 10 November 2006 (1♀), R. Segura & M. Martínez, LPL 10926. El Caracol, 22° 09.6′ N, 98° 01.9′ W, 10 November 2006 (1♂), R. Segura & M. Martínez, LPL 10912. Lázaro Cárdenas, 22° 15.7′ N, 98° 8.73′ W, 17 May 2006 (1♂), M. Martínez & C. Pascacio, LPL 10657. Ciudad Cuauhtémoc, 22° 12.8′ N, 97° 53.8′ W, 17 May 2006 (1♂), R. Segura & C. Pascacio, LPL 10664.

***Platynota helianthes* (Meyrick) (Figures 5, 6)**
*Platynota subargentea* ranges from Mexico to Venezuela (based on specimens in USNM). The male is similar to *P. rostrana,* with a complex hood on the frons and a long forewing costal fold; but the two species are easily distinguished by features of the genitalia, in particular those of the female.
**Biology.** This species has been reared from *Jatropha gossypifolia* L. (Euphorbiaceae), *Casearia corymbosa* H. B. & K. (Flacourtiaceae), *Leucania leucocephala* (Lam.) De Wit (Leguminosae), *Mimosa pigra* (Leguminosae), and *Psidium guajava* L. (Myrtaceae) in Mexico (all USNM). We reared *P. helianthes* from *Parkinsonia aculeata* in Nicaragua and Mexico.
**Specimens Examined.** MEXICO: San Luis Potosí: La Marland 2, 22° 10.1′ N, 98° 24.4′ W, 1 March 2008 (1♀), R. Segura, M. Martínez & C. Pascacio, LPL 11587. Veracruz: Canoas, 22° 10.1′ N, 98° 07.7′ W, 16 August 2002 (2♂, 3♀), M. Martínez & C. Pascacio, LPL 12054, 12058, 12062, 12064. NICARAGUA: Matagalpa, Las Guayabas 1, 11 December 1999 (1♀), T. Heard & R. Segura, LPL 8839.

***Platynota stultana* Walsingham (Figures 7, 8)**
*Platynota stultana* is one of the smaller species in the genus. It ranges throughout the southwestern United States and northern Mexico; it is adventive in California (Powell 1983), Hawaii (Miller and Hodges 1995), Florida (Kimball 1965), Virginia, and Washington, D.C. (USNM).
**Biology.** This species is known in the American economic literature as the omnivorous “leaf-roller”. Larval hosts in native situations include western ragweed (*Ambrosia psilostachya* DC; Asteraceae), gooseberry (*Ribes* sp.; Grossulariaceae), and youngberries (*Rubus* sp.; Rosaceae). Agricultural and ornamental hosts include a wide range of native and cultivated plants (Atkins et al. 1957; MacKay 1962; Powell 1983; Miller and Hodges 1995). Rolled leaves of *Parkinsonia aculeata* harboring larvae of *Platynota stultana* were collected at several sites in a large swamp known as Laguna Ajinche near the town of Ebano, San Luis Potosí, Mexico, on 10 November 2006. First instars fed inside the pinnule and soft rachis of the host, eventually feeding externally, tying the young leaves together with silk to make a shelter from which they foraged on young leaves. In a host specificity test, oviposition, development, and adult emergence of *P. stultana* occurred on all test plant species (*Mimosa asperata* L., *Leucaena leucocephala* (Lam.) de Wit, *Caesalpinia pulcherrima* (L.) Sw., *Delonix regia* (Bojer ex Hook.) Raf, *Tamarindus indica* L., and *Parkinsonia texana*) (all Leguminosae).
**Specimens Examined.** MEXICO: San Luis Potosí: Pozo La Pez No. 1, 22° 5′ N, 98° 23.4′ W, 10 November 2006 (13♂, 3♀), R. Segura & M. Martínez, LPL 10937, 10940, 10941, 10943, 10945–10950. Playa Buda, 22° 10.8′ N, 98° 22.8′ W, 10 November 2006 (1♂, 2♀), R. Segura & M. Martínez, LPL 10927, 10929. La Marland, 22° 08.4′ N, 98° 24.9′ W, 9 November 2006 (1♂), R. Segura & M. Martínez, LPL 10896. Veracruz: El Caracol, 22° 9.6′ N, 99° 1.9′ W, 10 November 2006 (1♂*,* 1♀), R Segura & M. Martínez, LPL 10605, 10909. Laguna Chairel, 22° 13.4′ N, 97° 52.9′ W, 16 May 2006 (1♂), R Segura & C. Pascacio, LPL 10605.

**Figure 9–14.**
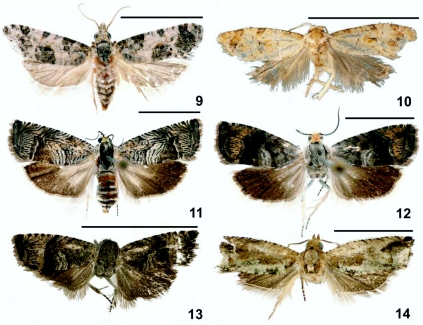
Adults of leaf-roller moths reared from *Parkinsonia aculeata.* 9) *Rudenia leguminana* (Busck). 10) *Cochylis* sp. 11) *Ofatulena duodecemstriata* (Walsingham). 12) *Ofatulena luminosa* Heinrich. 13) *Ofatulena* n. sp. 14) *Crocidosema lantana* Busck. Scale bar = 5 mm. High quality figures are available online.

**Figure 15.**
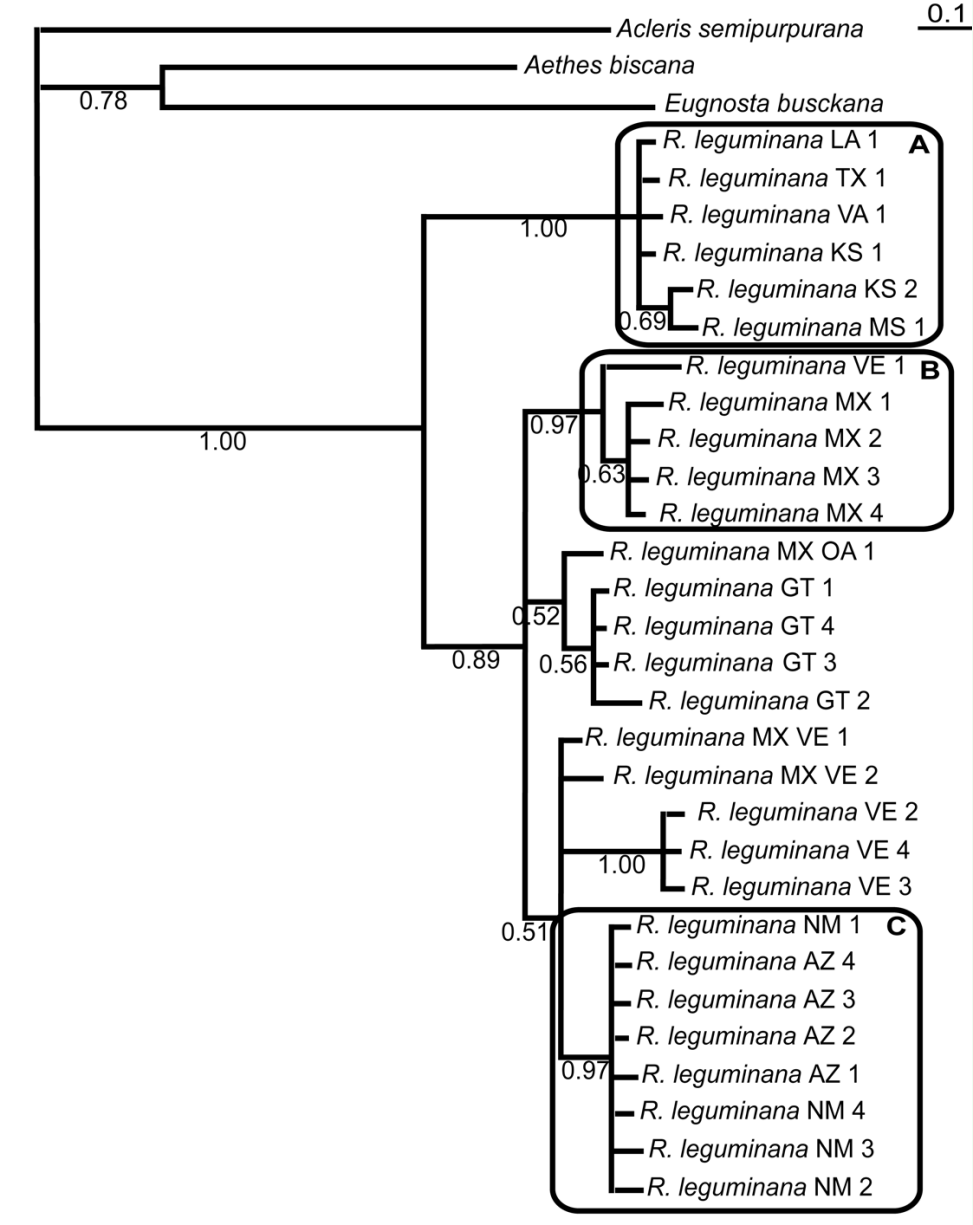
Bayesian tree based on Data Set II. Numbers below branches are posterior probabilities. Circled clades (A, B, and C) are postulated to be different species. Abbreviations are as follows: USPS abbreviation for the U.S. states, Guatemala (GT), Mexico (MX), Mexico Oaxaca (MX, OA), Mexico Veracruz (MX, VE), and VE (Venezuela). High quality figures are available online.

***Rudenia leguminana* (Busck) complex (Figure 9)**
*Rudenia leguminana* is widely distributed in North America, ranging from southern Connecticut to Florida, and from the midwestern U.S. to California. It is abundant in some xeric areas of the southwestern U.S. (i.e. Texas, New Mexico, Arizona) where *Prosopis* sp. (mesquite) and/or *Acacia* spp. (both Leguminosae) are the dominant shrubs.
**Biology.** Based on specimen label data and one published record (i.e., Busck 1907), this species has been reared from *Acacia farnesiana* (L.) Willd. (USNM), *A. glauca* (L.) Moench (USNM), *A. novernicosa* Isley (USNM), *Gleditsia japonica* Micq. (Busck 1907), *Leucaena pulverulenta* (Schltdl.) Benth. (USNM), *Mimosa aculeaticarpa* var. *biuncifera* (Benth.) Barneby (USNM), *Prosopis glandulosa* Torr. (USNM), and *Senna lindheimeriana* (Scheele) H. S. Irwin & Barneby (USNM) (all Leguminosae).
*Rudenia leguminana* was reared from *P. aculeata* in Guatemala (Jutiapa), Mexico (San Luis Potosí, Oaxaca, and Veracruz), Nicaragua (Granada, Managua, and Matagalpa), and Venezuela (Miranda and Anzoátegui), and from *Pithecellobium dulce* (Leguminosae) in Mexico (San Luis Potosí). On *P. aculeata* eggs are laid on leaves (rachis and pinnules) and tips of other vegetative parts. First instars feed inside the rachis. They then move to the axil and make a tunnel within it. Larvae leave the tunnel at night to feed on the pinnules and rachis of leaves. Frass can be seen protruding from tunnels especially during the mid-life of larvae when the tunnel is being actively excavated. Pupation usually occurs in tunnels, and when adults emerge the exuvium is partly exuded from the tunnel. Adults of *Rudenia leguminana* emerged from stems (n = 417 individuals), flowers (n = 90), and pods (n = 29) of the host.
**Host Specificity Trials.** The large number of individuals of *R. leguminana* reared from *P. aculeata* in the field indicated that this herbivore was worthy of further consideration as a biocontrol agent, so additional studies were conducted. “No-choice” host specificity trials were performed in 2006. Three females and 4 males were placed in bags covering tips of 6 test plant species and the control, *P. aculeata* (Table 1). Successful development on 5 non-target hosts resulted: *Parkinsonia praecox* (Ruiz & Pav.) J. A. Hawkins, *Mimosa asperata* L., *Mimosa pigra, Delonix regia,* and *Acacia farnesiana.* However, oviposition, development, and adult emergence of *R. leguminana* on non-target hosts may have been an artifact of the confined conditions of the trials. For example, *R. leguminana* were never reared from *Mimosa pigra* in the field despite considerable research on the herbivores of this plant over many years in the same geographic region.
  To better establish the field host range, an open-field trial was conducted in a plot at La Aguada, Veracruz, Mexico. Eleven plant species were grown in a plot: *P. aculeata, Mimosa asperata, Mimosa pigra, Mimosa p-dica* L., *Acacia farnesiana, Caesalpinia pulcherrima, Calliandra grandiflora* (L'Her.) Benth., *Desmanthus virgatus* (L.) Willd., *Inga jinicuil* G. Don, *Leucaena leucocephala,* and *Tamarindus indica* (all Leguminosae). Two releases of lab-reared adults were made; the first consisted of 42 females and 42 males on 24 July 2006 and the second of 52 females and 52 males on 9 August 2006. When damage to tips of the plants was observed, the terminal area was bagged to capture emerging adults. In these trials, *R. leguminana* successfully developed only on *P. aculeata* (20 from the first trial and 34 from the second). Although *R. leguminana* successfully developed on *Mimosa asperata, Mimosa pigra,* and *Acacia farnesiana* in the no-choice trials, no feeding or adult emergence was detected on these plant species in the open-field trials. This suggests that if not confined with the non-target hosts, *R. leguminana* may not oviposit on them, or at least development is far less likely.
**Molecular Analyses.** Because few native tortricids range from the northeastern U.S. to Venezuela, it is possible that more than one species is concealed within the material examined. Razowski (1985) indicated that specimens from Sonora, Sinaloa, and Baja California have genitalia very similar to “*R. leguminana* and may be conspecific with that species,” even though they exhibit some external and genital variation. Although some variation (in facies and genitalia) is present in the material reared from *P. aculeata,* the variation does not co-vary and is not concordant with geography.
  Hence, four genetic markers were analyzed to see if molecular data could differentiate groups within this broad geographic distribution. In one data set (Data Set I) two mitochondrial markers were examined, a 781-basepair region of cytochrome c oxidase I (COI) and a 685-basepair region of cytochrome c oxidase II (COII), and a 531-basepair region of the nuclear marker 28S domain 2 (D2). In Data Set I 5 individuals were sampled from Mexico, 3 from Nicaragua, 1 from Arizona, and 1 from Nebraska. In addition, another data set (Data Set II) was analyzed which consisted of the barcode region of COI. In Data Set II 29 individuals of *Rudenia leguminana* were sampled ranging from Virginia to Venezuela, and 3 other tortricids as outgroups (*Acleris semipurpurana, Aethes biscana,* and *Eugnosta* *busckana*). Evolutionary pairwise distances in PAUP* 4.0b10 (Swofford 2002) were calculated using a maximum likelihood model with parameter estimates from a maximum likelihood analysis carried out in Garli 0.951 (Zwickl 2006) with the GTR+I+G model, selected as best by Modeltest 3.7 (Posada and Crandall 1998) based on the AIC criterion. A tree based on Data Set II was inferred using MrBayes 3.1.2 (Huelsenbeck and Ronquist 2001) with a data set partitioned by codon position into 3 subsets, with uninformative priors except for the branch length prior, 5 million generations (burnin=2 million), 4 chains (1 cold, 3 heated), and 2 concurrent runs. For establishing branch length prior, the empirical Bayes method was employed, calculating mean branch length for the maximum likelihood tree from 4 separate Garli analyses and using the resulting value for creating an exponential distribution for this prior. Whether the MCMC chains converged was assessed by examining the potential scale reduction factors and log-likelihood plots over time.
  Evolutionary distances between individuals in both Data Set I and Data Set II strongly suggest that these individuals belong to more than one species. In Data Set I, evolutionary distances for individuals from the same geographic area are at the level expected for conspecifics (below 1% for both mitochondrial markers) (Hebert et al. 2003), whereas distances for individuals from more distant locations show a divergence an order of magnitude greater (Tables 2 and 3). There is very little variation in D2. Based on the analysis of the Data Set II (COI barcode) it appears that there are at least three different species (Figure 15), with distances within species ranging 0.0–2.3% and between species 4.9–13% (Table 4). Divergence levels of greater than 3% frequently are interpreted to represent separate species (Hebert et al. 2003; Hebert et al. 2004). It is odd that one of the individuals from Venezuela (VE 1) is about 5% distant from the three other Venezuelan specimens (however, it is possible that this is the result of contamination). Also, the origin of the four specimens from Mexico (MX 1–4) that cluster with the Venezuelan outlier is uncertain because these specimens are larvae that were intercepted at U.S. ports-of-entry from “Mexico” on *Pithecellobium dulce* (i.e. the exact point of origin is unknown, although it is likely to be northern Mexico).
  Although geographic sampling is limited, these results strongly suggest that more than one species is present in the material examined: species A, including individuals from the eastern U.S. (e.g. Kansas, Louisiana, Mississippi, Texas, and Virginia); species B, including individuals from Mexico (Oaxaca and Veracruz) and Venezuela; and species C, including individuals from southwestern U.S. (Arizona and New Mexico) (Figure 15). Owing to weak support for the rest of the tree, we refrain from drawing conclusions regarding the assignment to species of the remaining individuals.
**Summary of Collecting Localities.** GUATEMALA: Jutiapa: Asunción Mita, Finca El Platanar, 14° 17.29′ N, 89° 33.05′ W, LPL 10952. El Guayabo, Lago Güija, 14° 13.9′ N, 89° 32′ W LPL 10958-967. El Platanar, El Chamizal, 14° 18.22′ N, 89° 36.96′ W LPL 10973, 10977, 10975, 10979–10980, 10981, 10982, 10983, 10984, 10986. El Platanar, Lago Güija, Laguna del Muerto, 14° 17.54′ N, 89° 34.33′ W, LPL 10998–11000, 100002–11004. MEXICO: Oaxaca: Ciudad Ixtepec, Base Aérea Militar No. 2, 16° 26.55′ N, 95° 4.65′ W, LPL 10524, 10829. Tehuantepec: Alvaro Obregón, 16° 20.9′ N, 95° 4.5′ W, LPL 9802–9803. Huazantlán del Río, 16° 12.7′ N, 95° 6′ W, LPL 9634, 9859, 10026–10033, 10081–10084, 10159–161, 10241–251, 10277–286, 10498, 10561, 10808, 11278–281. El Jordan, 16° 22.1′ N, 95° 12.8′ W, LPL 12227–12230, 12441–12444. San Luis Potosí: Lago Ajinche, La Marland, 22° 8.46′ N, 98° 24.98′ W, LPL 10861, 10865, 12446. Laguna Ajinche, 22° 10.5′ N, 98° 21.0′ W, LPL 12396, 12412, 12415, 12440. Laguna Chica, 22° 11.1′ N, 98° 23.0′ W, LPL 12399, 12411, 12416. Laguna Chica, Playa Buda, 22° 10.85′ N, 98° 22.88′ W, LPL 11129, 11169. La Muralla, 22° 11.8′ N, 98° 20′ W, LPL 12413, 12419, 12420, 12428, 12450. La Muralla, 22° 11.8′ N, 98° 20′ W, LPL 12418, 12408, r.f. *Pithecellobium dulce.* Pozo La Pez No. 1, 22° 10.5′ N, 98° 23.4′ W, LPL 12398, 12447. Veracruz: Cacalilao, Lazaro Cárdenas, 22° 15.79′ N, 98° 8.73′ W, LPL 10656. Veracruz: Canoas, 22° 10.1′ N, 98° 07.7′ W, LPL 12207, 12414, 12426, 12451, 12454. Tamos, El Caracol, 22° 9.63′ N, 98° 1.92′ W, LPL 11154–157, 11193, 12208, 12224, 12397, 12409, 12417, 12422, 12457, 12458, 12459. Tamos, La Cortadura, 22° 10.98′ N, 98° 1.36′ W, LPL 10849, 10853, 10905, 12410, 12420, 12427, 12461. NICARAGUA: Granada: La Playuela, 12° 2.8′ N, 85° 55.3′ W, LPL 9816, 10041–47, 10095. Laguna La Playuela 1, 12° 1.8′ N, 85° 55.3′ W, LPL 9734, 9737–9738, 9740, 9749. Laguna La Playuela 2, 12° 1.3′ N, 85° 55.2′ W, LPL 9748–9750, 9760. Managua: road to San Francisco Libre, km 74, 12° 22.6′ N, 86° 6.7′ W, LPL 9861. Matagalpa: Tecomapa, 12° 37.6′ N, 86° 2.2′ W, LPL 9869–70. La Playuela, 12° 2′ N, 85° 56.3′ W, LPL 9871–81. Las Guayabas, 12° 37.7′ N, 86° 17′ W, LPL 8812, 8814, 8816–8817, 8819, 8841, 8842. VENEZUELA: Anzoategui, hwy Barcelona-Caracas, km 18, 10° 3.34′ N, 64° 48.31′ W, LPL 11079, 11081, hwy Barcelona-Caracas, km 11, 10° 2.93′ N, 64° 44.56′ W, LPL 11083. Miranda, La Guira, Mare Abajo, 10° 36.82′ N, 67° 1.23′ W, LPL 11045.

**Table 1.**
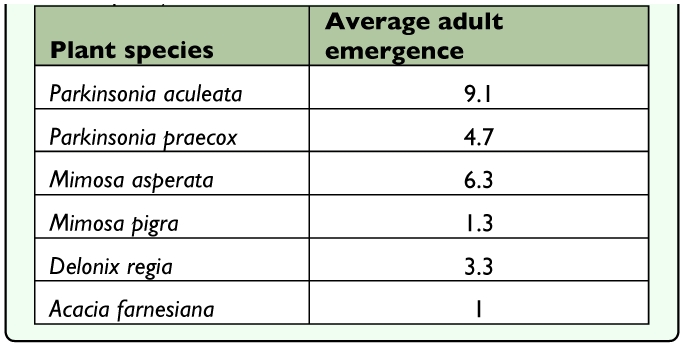
Adult emergence of “no choice” host specificity trials of *Rudenia leguminana* from six plant species (as recorded per individual plant).

**Table 2.**
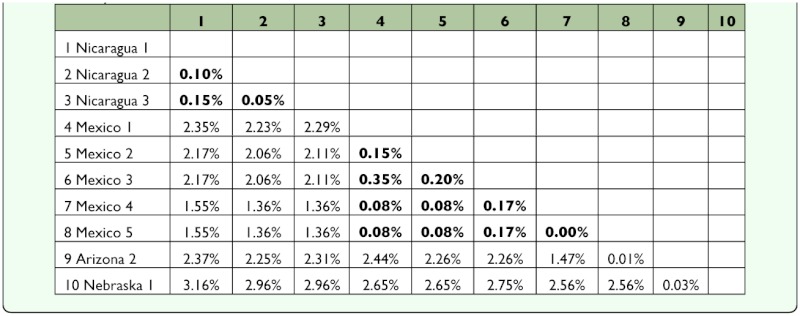
Evolutionary distances among individuals from Data Set I for the COI marker. Distances between individuals from the same locality are in bold.

**Table 3.**
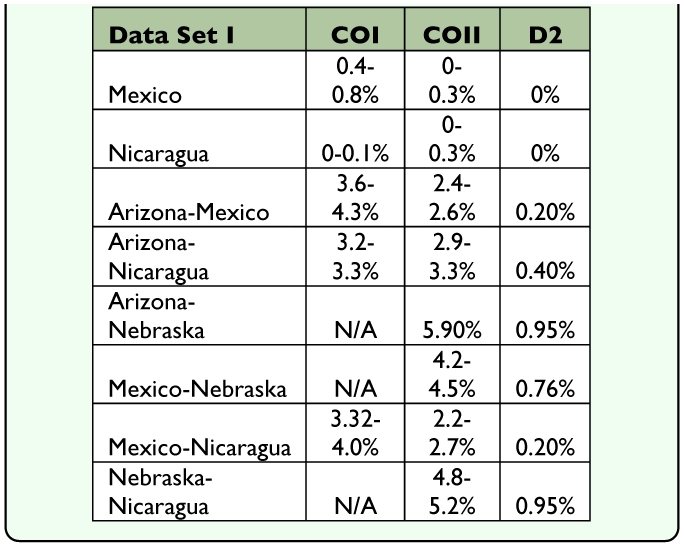
Evolutionary distances between individuals from the same area and between groups from more distant areas in Data Set I.

**Table 4.**
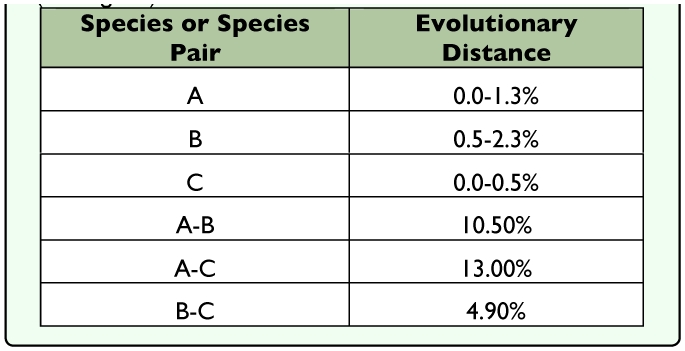
Evolutionary distances within and among species A, B, C (see Fig. 15) in Data Set II.

***Cochylis* sp. (Figure 10)**
Two females of an undetermined species of *Cochylis* were reared from Veracruz, Mexico. The taxonomic disarray of the group and the large number of undescribed taxa combine to inhibit accurate species-level identification.
**Biology.** Although most Cochylini worldwide feed on Asteraceae (Razowski 1970), there are numerous deviations from this pattern, and Leguminosae is not an unusual host plant family for members of this tribe.
**Specimens Examined.** MEXICO: Veracruz: La Cortadura, 22° 10.9′ N, 98° 1.36′ W, 14 April 2007 (1♀), R. Segura, T. Heard, M. Martínez, C. Pascacio, LPL 11152. San Luis Potosí: Playa Buda, 22° 9.63′ N, 98° 1.92′ W, 14 April 2007 (1♀), R. Segura, T. Heard, M. Martínez, C. Pascacio, LPL 11168.

***Ofatulena* Heinrich, 1926**
As defined by Heinrich (1926), *Ofatulena,* included two species: *O. duodecemstriata* (Walsingham) and *O. luminosa* Heinrich. The two share a whitish gray, finely striated forewing with a distinct ocellar patch in the tornal region; as in most Grapholitini, males of *Ofatulena* lack a costal fold. The male genitalia (illustrated by Heinrich 1926) have a distally swollen cucullus with a row of long, stout, flattened, marginal spiniform setae along the outer portion of the valva. “*Enarmonia*” Walsingham was transferred by Razowski to *Ofatulena* without comment in the checklist of Neotropical Lepidoptera (Powell et al. 1995). However, because it lacks the typical forewing pattern and shape, and the male genitalia deviate considerably from those of *O. duodecemstriata* (Walsingham) and *O. luminosa* Heinrich, we remove it from the genus and transfer it provisionally to *Cydia* (new combination), with which the facies and genitalia are more similar. *Ofatulena duodecemstriata* usually can be distinguished superficially from *O. luminosa* by its slightly greater forewing length and absence of peach or orange scaling in the forewing ocellar patch. The male genitalia of *O. duodecemstriata* have a linear patch of larger spiniform setae along the lower edge of the valva subapically and an extra, stout spiniform seta near the middle of the apical region. In *O. luminosa* the setae are more numerous, but much finer and thinner, and usually are easily dislodged in slide-mounted preparations. Specimens reared from *Parkinsonia praecox* in Oaxaca, Mexico apparently represent a third and undescribed species of *Ofatulena.* Based on collection records (USNM) and published literature (Heinrich 1926; MacKay 1959; Woods 1992), *Ofatulena* has been reared only from Leguminosae.

***Ofatulena duodecemstriata* (Walsingham) (Figure 11)**
*Ofatulena duodecemstriata* ranges across the western United States from California to Texas, north to Utah and south into Mexico. In addition, a series of this species (n = 6) was discovered in the USNM with the following data: Venezuela, Lara, Puente Torres, 24 km E Carora, 10 March 1978, thorn forest, blacklight, J. B. Heppner. The latter represent a considerable increase in the previously documented geographic range of the species. Although the Venezuelan specimens are considerably smaller than those from North America, the genitalia are identical to North American specimens.
**Biology.** The primary larval host in North America is mesquite (“mesquite beans”), *Prosopis* sp. (Leguminosae) (Heinrich 1926; MacKay 1959). Although there is a specimen in the USNM reared from *Verbascum thapsus* L. (Scrophulariaceae), this is almost certainly an error. One specimen from *P. aculeata* was reared in Mexico and two in Venezuela.
**Specimens Examined.** MEXICO: Oaxaca: Tehuantepec, 16° 17.5′ N, 95° 13.8′ W, 5 February 2002 (1♂), M. Martínez & M. Juárez, LPL 9622. VENEZUELA: Nueva Esparta: Bahía de Plata, 11° 6.47′ N, 63° 56.8′ W, 12 March 2007 (1♂, 1♀), R. Segura & C. Pascacio, LPL 11060.

***Ofatulena luminosa* Heinrich (Figure 12)**
This species is recorded from California (EME), Arizona (Woods 1992), and Texas (USNM, ANIC), USA, and Sonora, Sinaloa, and Nuevo Leon, Mexico (ANIC). Larvae have been reported previously only from *P. aculeata* (Woods 1992). According to Woods (1992), “Up to 5 larvae have been dissected from the distal 25 cm of a stem [of *P. aculeata*]. A single larva may also develop in a green seed, eating out the seed and killing it. Heavily infested plants appear to grow at a slower rate than uninfested ones.” Woods (1992) also mentions that the larvae of *O. luminosa* are heavily parasitized by Hymenoptera.
**Biology.** Specimens of *O. luminosa* were reared from *P. aculeata* in Oaxaca, San Luis Potosí, and Veracruz, Mexico and from *P. texana* in San Luis Potosí. The abundance of individuals in the field and the damage it causes indicated that this herbivore could have potential as a biocontrol agent, so additional observations and studies were conducted.
  Larvae bore in leaf and stem tips, mature green stems, and green seeds. The stem may appear swollen as a result of the presence of the larvae. Most damage is concentrated in the pith in the center of the stems. When mature, the larva prepares an exit hole distinguished by a frass and silk structure. From 218 bagged stems which showed evidence of damage, 96 adults emerged. Two to 3 damaged stems were observed per plant on 2 transects showing that this insect is consistently and abundantly available (Table 5). Overall, adults of *Ofatulena luminosa* emerged from stems (n = 164 individuals), pods (n = 55), and flowers (n = 2) of the host. Adults live for a mean of 6 days.
**Surveys of Natural Host Plant** Use. Twenty-three legume species growing in the same habitat as *P. aculeata* were recognized (5 species of Caesalpinioideae, 11 species of Mimosoideae, and 7 species of Faboideae) (Table 6). Stems with evidence of damage were bagged for adult emergence. In October 2008 when the site was threatened with flooding, all stems were collected and taken to the lab where they were held in plastic bottles until insects emerged, or were dissected if the stem showed signs of drying out. All insects that emerged were pinned, labeled, and identified. *Ofatulena luminosa* emerged only from *Parkinsonia aculeata* and *P. texana* (A. Gray) S. Watson var. *macra* (I.M. Johnst.) Isely.
**Specimens Examined.** MEXICO: Oaxaca: Comitancillo, 16° 28.8′ N, 95° 5.5′ W, 18 August 2009 (1♀), M. Martínez & C. Pascacio, LPL 12537. Huazantlan del Rio, 16° 12.7′ N, 95° 6′ W, 19 August 2009 (6♂, 8♀), M. Martínez & C. Pascacio, LPL 12546. San José del Palmar, 16° 13.84′ N, 95° 10.77′ W, 19 August 2009 (1♂), M. Martínez & C. Pascacio, LPL 12550. San Luis Potosí: Laguna Ajinché, 22° 10.5′ N, 98° 21.0′ W, 18 May 2006 (12♂, 5♀), R. Segura & C. Pascacio, LPL 10681, 10690, 10691, 23 April 2009 (1♂, 1♀), M. Martínez & C. Pascacio, LPL 12404, 12464. Ajinché, 22° 11.8′ N, 98° 21.1′ W, 16 May 2006 (1♂, 1♀), R. Segura & C. Pascacio, LPL 10644. Laguna Chica, 22° 11.1′ N, 98° 23.0′ W, 19 May 2008 (1♂, 1♀), M. Martínez, LPL 11725, 11742, 21 April 2009 (1♂), R. Segura, M. Martínez, C. Pascacio, T. Heard, LPL 12424. La Marland, 22° 11.8′ N, 98° 24.9′ W, 8 November 2006 (1♀), Segura & Martínez, LPL 10866, 13 April 2007 (3♂, 2♀), R. Segura, M. Martínez, T. Heard, C. Pascacio, LPL 11147, 11148, 21 April 2009 (1♀), Segura, Martínez, Pascacio & Heard, LPL 12423. La Marland 2, 22° 08.4′ N, 98° 24.9′ W, 13 January 2009 (1♂), M. Martínez & C. Pascacio, LPL 12219. Pozo La Pez No. 1, 22° 10.5′ N, 98° 23.4′ W, 13 April 2007 (1♂, 1♀), Segura, Martínez, Pascacio & Heard, LPL 11118, 21 April 2009 (1♀), Segura, Martínez, Pascacio & Heard, LPL 12403. La Muralla, 22° 11.8′ N, 98° 20′ W, 18 May 2008 (5 specimens), LPL 11683, 11684, 11685, 11721, 11722, 21 May 2008 (5 specimens), LPL 11687, 11688, 11689, 11690, 11744, 13 January 2009 (1♀), M. Martínez & C. Pascacio, LPL 12212, 22 April 2009 (1♂), M. Martínez & C. Pascacio, LPL 12462, 21 April 2009 (1♂, 1♀), Segura, Martínez, Pascacio & Heard, LPL 12402, 12449. Veracruz: Canoas, 22° 10.1′ N, 98° 07.7′ W, 10 November 2006 (1♀), Segura & Martínez, LPL 10678, 10 November 2006 (1♀), R. Segura & C. Pascacio, LPL 10919, 16 August 2008 (1♂), Martínez, LPL 12191, 22 April 2009 (2♂, 1♀), Segura, Martínez, Pascacio & Heard, LPL 12401, 12452, 12455, 14 September 2008 (1♀), C. Pascacio, LPL 12197, 17 September 2008 (1♂, 1♀), Segura & Pascacio, LPL 12200. Ciudad Cuauhtemoc, 22° 12.8′ N, 97° 53.8′ W, 17 May 2006 (2♂), R. Segura & C. Pascacio, LPL 10663. El Caracol, 22° 09.6′ N, 98° 01.9′ W, 10 November 2006 (3♂, 3♀), R. Segura & M. Martínez, LPL 10913, 10910, 10911, 22 April 2009 (2♂, 1♀), Segura, Martínez, Pascacio & Heard, LPL 12456, 12460. El Caracol, 22° 09.3′ N, 98° 02.4′ W, 16 August 2008 (1♂), M. Martínez & C. Pascacio, LPL 12192. La Cortadura, 22° 10.9′ N, 98° 01.3′ W, 8 November 2006 (1♂), R. Segura & M. Martínez, LPL 10855, 22 April 2009 (1♀), Segura, Martínez, Pascacio & Heard, LPL 12425. Cardenas, 22° 15.7′ N, 98° 8.73′ W, 17 May 2006 (1♀), R. Segura & M. Martínez, LPL 10650.

**Table 5.**
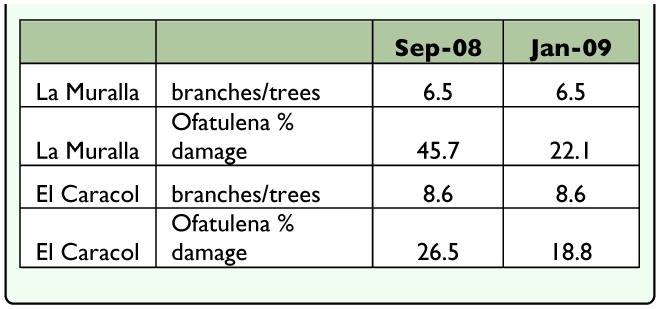
Density of *O*. *luminosa* larval damage in the field at La Muralla and El Caracol.

**Table 6.**
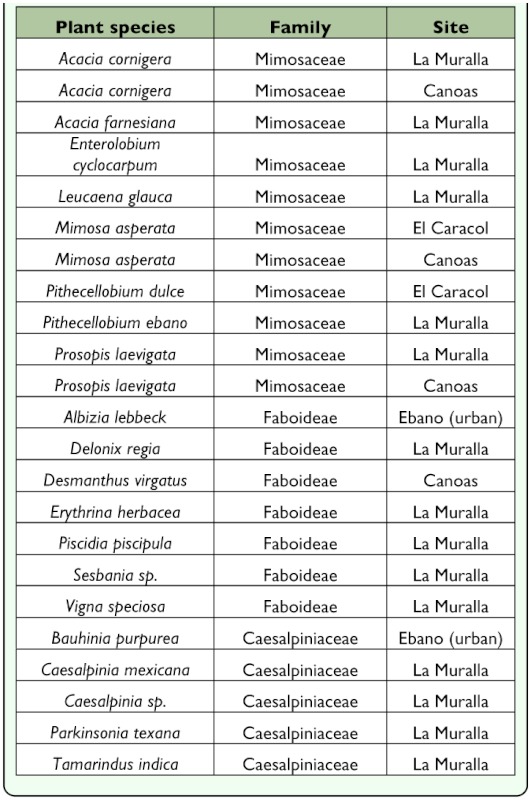
Related legumes growing in the same habitat as *Parkinsonia.*

***Ofatulena* sp. (Figure 13)**
Two specimens of *Ofatulena* were reared from *Parkinsonia praecox* in Oaxaca, Mexico. The adults have a forewing length conspicuously less than that of either *O. duodecemstriata* or *O. luminosa,* the forewing has slightly darker scaling, and the third segment of the labial palpus is brown (white in the other two species). In the male genitalia (n = 1), the swollen portion of the valva is less inflated, and the flattened scales in a row along the perimeter of the valva are somewhat uniformly spaced and easily dislodged. The two specimens likely represent an undescribed species.
**Biology.** Apart from the host, nothing is known of the biology of this apparently undescribed species.
**Specimens Examined.** MEXICO: Oaxaca: El Jordan, 16° 24.3′ N, 98° 11.6′ W, 21 January 2009 (1♂, 1♀), M. Martínez & C. Pascacio, LPL 12435, 12231.

***Crocidosema lantana* Busck (Figure 14)**
*Crocidosema lantana* is indigenous to Mexico; it is recorded from Florida to Costa Rica. It was described from Hawaii (Busck 1910) based on specimens imported from Mexico for biological control of weedy lantana (*Lanana camara* L.: Verbenaceae) (Perkins and Sweezy 1924; Zimmerman 1978). It subsequently was introduced into Australia (Tyron 1914; Common 1957) and Miocronesia (Zimmerman 1978) for biological control.
**Biology.** This species has been reared from *Tabebuia chrysantha* (Jacq.) G. Nicholson (Bignoniaceae), *Tecoma stans* (L.) Juss. ex Kunth (Bignoniaceae), *Lantana camara* L. (Verbenaceae), and *Litchi chinensis* Sonn. (Sapindaceae) (Busck 1910; Tyron 1914; Perkins & Swezey 1924; Common 1957; MacKay 1959; Kimball 1965; Clarke 1976; Zimmerman 1978, Ibrahim & Zakaria 1988; Muniappan 1990). A single female of *C. lantana* was reared from *Parkinsonina aculeata* in San Luis Potosí, Mexico.
**Specimen Examined.** MEXICO: San Luis Potosí: La Muralla, 22° 11.8′ N, 98° 20′ W, 13 January 2009 (1♀), M. Martinez & C. Pascacio, LPL 12210.

## Concluding Remarks

The ten species of Tortricidae reared from *P. aculeata* in this study show a range of feeding modes from leaf rollers to borers in stems and reproductive parts. Their systematic positions vary from clearly defined species to possible species complexes, and include members of both of the largest subfamilies — Tortricinae and Olethreutinae. Their host specificity also varies widely from extreme generalists to possible specialists on the genus *Parkinsonia.* Although 7 of the 10 species are clearly not suitable as biocontrol agents, 2 species show some potential. *Rudenia leguminana* is probably a complex of species, one or more of which may be a specialist. Further work is needed to understand the systematics of this group. *Ofatulena leguminosa* is possibly a specialist on the genus *Parkinsonia.* This level of specificity may be suitable for the importation of this species into Australia as no other species of *Parkinsonia* occur there. Further work is ongoing to determine its host specificity and its impact on the target plant.

## References

[bibr01] Atkins EL, Frost MH, Anderson LD, Deal AS (1957). The “omnivorous leaf roller”, *Platynota stultana* Wlshm., on cotton in California: nomenclature, life history, and bionomics 4 (Lepidoptera: Tortricidae).. *Annals of the Entomological Society of America*.

[bibr02] Bailey FM (1906).

[bibr03] Bruner SC, Scaramuza LC, Otero AR (1975). *Catálogo de los insectos que atacan a las plantas económicas de Cuba: segunda edición revisada y aumentada.*.

[bibr04] Busck A (1907). A review of the tortricid subfamily Phaloniinae with descriptions of new American species.. *Journal of the New York Entomological Society*.

[bibr05] Busck A (1910). New Central American Microlepidoptera introduced into the Hawaiian Islands.. *Proceedings of the Entomological Society of Washington*.

[bibr06] Clarke JFG (1976). Microlepidoptera: Tortricoidea.. *Insects of Micronesia*.

[bibr07] Common IFB (1957). The occurrence of *Epinotia lantana* (Busck) (Lepidoptera: Olethreutidae) in Australia.. *Proceedings of the Linnean Society of New South Wales*.

[bibr08] d'Araujo Silva AG, Goncalves CR, Galvao DM, Goncalves AJL, Gomes J, do Nascimento Silva M., de Simoni L (1968). *Quarto catalogo do insetos que vivem nas plantas do Brasil.* Parte II-1 Tomo. Insetos, hospedeiros e inimigos narurais..

[bibr09] Diniz IR, Morais HC (1995). Larvas de Lepidoptera e sua plantas hospedeiras em um cerrado de Brasilia, D. F., Brazil.. *Revista Brasileira de Entomologia*.

[bibr10] Hawkins JA, Boutaoui N, Cheung KY, van Klinken RD, Hughes CE (2007). Intercontinental dispersal prior to human translocations revealed in a cryptogenic invasive plant.. *New Phytologist*.

[bibr11] Heard TA, Preston C, Watts JH, Crossman ND (2006). *Parkinsonia aculeata*: surveys for natural enemies, native range ecological studies and prospects for biological control.. *Proceedings of the 15th Australian Weeds Conference*.

[bibr12] Hebert PDN, Ratnasingham S, deWaard JR (2003). Barcoding animal life: cytochrome c oxidase subunit 1 divergences among closely related species.. *Proceedings of the Royal Society B*.

[bibr13] Hebert PDN, Penton EH, Burns J, Janzen DH, Hallwachs W (2004). Ten species in one: DNA barcoding reveals cryptic species in the neotropical skipper butterfly, *Astraptes fulgerator.*. *Proceedings of the National Academy of Sciences USA*.

[bibr14] Heinrich C (1921). Some Lepidoptera likely to be confused with the pink bollworm.. *Journal of Agricultural Research*.

[bibr15] Heinrich C (1926). Revision of the North American moths of the subfamilies Laspeyresiinae and Olethreutinae.. *United States National Museum Bulletin*.

[bibr16] Heinrich C (1931). Notes on and descriptions of some American moths.. Proceedings of the U.S. National Museum.

[bibr17] Huelsenbeck JP, Ronquist F (2001). MRBAYES: Bayesian inference of phylogeny.. *Bioinformatics*.

[bibr18] Ibrahim R, Zakaria M (1988). Insects associated with common weeds, special reference to fruit flies.. *Malaysian Applied Biology*.

[bibr19] Kimball CP (1965). The Lepidoptera of Florida: an annotated checklist.. Arthropods of Florida and neighboring land areas.

[bibr20] Liceras Z, Castillo J (1994). Nota preliminar sobre las plagas insectiles de la alfalfa en Huanchaco, Trujillo.. *Revista Peruana de Entomologia*.

[bibr21] Liljesthrom GG, Rojas GC, Pereyra PC (2001). Utilizacion de recursos y supervivencia larval del barrenador del brote, *Crocidosema aporema* (Lepidoptera: Tortricidae), en soja (*Glycine max*).. *Ecologia Austal*.

[bibr22] McClay AS, Palmer WA, Bennett FD, Pullen KR (1995). Phytophagous arthropods associated with *Parthenium hysterophorus* (Asteraceae) in North America.. *Annual review of Entomology*.

[bibr23] MacKay MR (1959). Larvae of the North American Olethreutidae (Lepidoptera).. *Canadian Entomologist Supplement*.

[bibr24] MacKay MR (1962). Larvae of the North American Tortricinae (Lepidoptera: Tortricinae).. *Canadian Entomologist Supplement*.

[bibr25] Miller SE, Hodges RW (1995). *Platynota stultana,* the omnivorous leaf-roller, established in the Hawaiian Islands
(Lepidoptera: Tortricidae).. *Bishop Museum Occasional Papers*.

[bibr26] Muniappan R (1990). Biological control of *Lantana camara* L. in Yap.. *Proceedings of the Hawaiian Entomological Society*.

[bibr27] Okumura GT (1974). Illustrated key to the identification of lepidopterous larvae attacking tomatoes in Mexico and the United States, excluding Alaska.. *National Pest Control Operators News*.

[bibr28] Pastrana JA (2004). Los Lepidopteros Argentinos, sus hospedadoras y otros sustratos alimenticios..

[bibr29] Perkins RCL, Swezey OH (1924). The introduction into Hawaii of insects that attack lantana.. *Bulletin of the Hawaii Sugar Association, Entomology,* Ser. 16..

[bibr30] Phillips-Rodriguez E, Powell JA (2007). Phylogenetic relationships, systematics, and biology of the species of *Amorbia* Clemens (Lepidoptera: Tortricidae: Sparganothini).. *Zootaxa*.

[bibr31] Posada D, Crandall KA (1998). Modeltest: testing the model of DNA substitution.. *Bioinformatics*.

[bibr32] Powell JA (1983). Expanding geographical and ecological range of *Platynota stultana* in California.. *Pan-Pacific Entomologist*.

[bibr33] Powell JA, Razowski J, Brown RL, Heppner JB (1995). Tortricidae: Olethreutinae.. *Atlas of Neotropical Lepidoptera, Checklist Part II: Hyblaeoidea - Pyraloidea — Tortricoidea*.

[bibr34] Razowski J, Amsel HG, Gregor F, Reiser H (1970). Cochylidae.. *Microlepidoptera Palaearctica*.

[bibr35] Razowski J (1985). Descriptions of *Rudenia* gen n. and its two new species (Lepidoptera, Tortricidae).. *Polskie Pismo Entomologiczne*.

[bibr36] Swofford DL (1998). PAUP*. *Phylogenetic analysis using parsimony (*and other methods). Version 4.*.

[bibr37] Tyron H (1914). Report of the entomologist and vegetable pathologist.. *Annual Report of the Department of Agriculture, Stock, Queensland*.

[bibr38] van Klinken R.D., Campbell S.D., Heard T.A., McKenzie J., March N (2009). The Biology of Australian Weeds *Parkinsonia aculeata* L.. *Plant Protection Quarterly*.

[bibr39] Woods W (1992). Phytophagous insects collected from *Parkinsonia aculeata* [Leguminosae: Caesalpiniaceae] in the Sonoran desert region of the southwestern United States and Mexico.. *Entomophaga*.

[bibr40] Zimmerman EC (1978). *Insects of Hawaii, Volume 9, Microlepidoptera,* Part 1..

[bibr41] Zwickl DJ (2006). *Genetic algorithm approaches for the phylogenetic analysis of large biological sequence datasets under the maximum likelihood criterion.*.

